# Modeling AI trust and AI self-efficacy in AI-assisted EFL classrooms: the role of basic psychological needs in classroom engagement

**DOI:** 10.3389/fpsyg.2026.1807643

**Published:** 2026-05-11

**Authors:** Huimin Guo, Mingyuan Zheng

**Affiliations:** School of Foreign Studies, North China University of Water Resources and Electric Power, Zhengzhou, China

**Keywords:** AI self-efficacy, AI trust, basic psychological needs satisfaction, classroom engagement, EFL

## Abstract

The increasing use of artificial intelligence (AI) in English as a foreign language(EFL) classroom has raised questions about how learners' perceptions of AI relate to their classroom engagement. Against this backdrop, this study examined the relationships among AI trust, AI self-efficacy, basic psychological needs (BPNs) satisfaction, and classroom engagement in an AI-assisted EFL context. A total of 1,002 Chinese university students completed a questionnaire measuring these constructs. Data were analyzed using confirmatory factor analysis and structural equation modeling (SEM). The results showed that both AI trust and AI self-efficacy were positively associated with learners' satisfaction of autonomy, competence, and relatedness. BPNs satisfaction, in turn, was strongly related to classroom engagement. However, the direct effects on classroom engagement were not significant. Instead, both variables showed significant indirect associations with engagement through BPNs satisfaction, indicating a pattern consistent with statistical mediation. These findings suggest that learners' AI-related beliefs are related to classroom engagement primarily through their associations with motivational experiences rather than directly driving engagement behaviors. This study contributes to understanding the relationships between learners' perceptions of AI and classroom engagement. The study also highlights the importance of need-supportive instructional design in AI-assisted EFL classrooms.

## 1 Introduction

The increasing integration of artificial intelligence (AI) into English as a foreign language (EFL) classrooms has reshaped how learners participate in and experience classroom learning. AI-based tools are now commonly used to support language practice, feedback, and task completion, making AI-supported activities a routine component of classroom instruction rather than an occasional supplement. However, previous research has consistently shown that the effectiveness of instructional innovations depends less on technological availability than on learners' active classroom engagement (Reeve, 2013; Skinner and Pitzer, 2012). In EFL contexts, where language development relies heavily on sustained participation and interaction, understanding learner engagement in AI-assisted classrooms has therefore become a critical issue.

Classroom engagement is widely conceptualized as learners' behavioral, emotional, and cognitive involvement in learning activities (Fredricks et al., 2004, 2016). In second language research, engagement has also been discussed as learners' engagement with language itself, emphasizing how learners attend to and work with linguistic input during classroom tasks (Svalberg, 2009). Accumulating evidence suggests that engagement is a dynamic and context-sensitive construct rather than a stable learner trait, reflecting learners' ongoing motivational experiences within specific instructional environments (Reeve, 2013; Zhang, 2021). From this perspective, classroom engagement serves as a meaningful indicator of learning quality in EFL classrooms, including those supported by AI technologies.

From a self-determination theory (SDT) perspective, classroom engagement is closely linked to learners' satisfaction of basic psychological needs (BPNs) for autonomy, competence, and relatedness (Deci and Ryan, 2000; Ryan and Deci, 2017). When these needs are supported, learners are more likely to invest effort, persist in learning tasks, and display positive learning-related emotions. Recent studies in AI-assisted EFL learning have suggested that instructional features such as flexible pacing, targeted feedback, and guided AI use may contribute to learners' perceptions of autonomy and competence, while supportive classroom integration of AI can foster a sense of relatedness (Annamalai et al., 2023; Chiu et al., 2023; Wang et al., 2025a). At the same time, poorly designed or overly controlling AI use may undermine these needs and weaken learners' engagement (Annamalai et al., 2023; Chiu et al., 2023).

In addition to motivational experiences, learners' beliefs about AI have been identified as important factors shaping AI-assisted learning. In EFL contexts, AI trust reflects learners' willingness to rely on AI systems for language-related support, whereas AI self-efficacy refers to learners' confidence in their ability to use AI tools effectively during learning tasks (Wang F. et al., 2025; Wang and Chuang, 2024). Emerging empirical evidence suggests that learners who trust AI and feel capable of using it are more likely to persist in AI-supported activities and perceive AI use as beneficial for learning (Annamalai et al., 2025; Chiu et al., 2024; Li and Chiu, 2025). However, these AI-related beliefs may not directly translate into sustained classroom engagement unless learners' BPNs are simultaneously satisfied (Ryan and Deci, 2017; Wang and Wang, 2024).

Against this background, the present study examines the relationships among AI trust, AI self-efficacy, BPNs satisfaction, and classroom engagement in AI-assisted EFL learning. By integrating SDT with research on AI-related beliefs, this study proposes and tests a structural model that includes both direct and indirect pathways linking learners' perceptions of AI to classroom engagement. In doing so, the study aims to clarify the motivational mechanisms through which AI-assisted instruction is related to learners' engagement and to provide empirical evidence to inform the pedagogical design of AI-supported EFL classrooms.

## 2 Literature review

### 2.1 Theoretical framework

Self-determination theory (SDT) provides an important theoretical framework for understanding learners' motivation and engagement in educational contexts. SDT posits that human motivation is shaped by the satisfaction of three basic psychological needs: autonomy, competence, and relatedness (Deci and Ryan, 2000). Autonomy refers to the experience of choice and psychological freedom in one's actions, competence reflects the sense of effectiveness and progress in learning tasks, and relatedness refers to the feeling of connection and support from others in a social environment (Ryan and Deci, 2000, 2017). When these needs are satisfied, individuals are more likely to experience intrinsic motivation, persistence, and active engagement in learning activities (Li et al., 2025).

Within educational settings, SDT has been widely used to explain why students become actively involved in learning. Research grounded in SDT suggests that when learning environments support students' autonomy, competence, and relatedness, learners are more likely to show greater behavioral participation, emotional involvement, and cognitive investment in classroom tasks (Noels, 2023; Wang and Xue, 2024). In contrast, when these needs are undermined, students' motivation and engagement tend to decline (Wang and Wang, 2024).

In technology-enhanced learning contexts, SDT has also been applied to understand how digital tools influence students' learning experiences. Previous studies indicate that technological environments can either support or hinder learners' psychological needs depending on how they are integrated into instructional practices (Li et al., 2025; Wu and Wang, 2025). In AI-assisted learning settings, features such as adaptive feedback, flexible learning support, and interactive assistance may contribute to learners' perceptions of autonomy and competence, while supportive teacher guidance and collaborative classroom use of AI can help maintain a sense of relatedness (Wang et al., 2025a; Wu and Wang, 2025). Based on this perspective, the present study adopts SDT as its guiding framework and examines whether AI trust and AI self-efficacy are associated with classroom engagement through the mediating role of basic psychological needs satisfaction.

### 2.2 Classroom engagement

Classroom engagement refers to students' active involvement in learning activities and can vary across time, tasks, and instructional contexts (Skinner and Pitzer, 2012). It is commonly viewed as a visible indicator of students' motivation, showing how much effort and persistence they invest in learning (Reeve, 2013). In EFL contexts, engagement is particularly important because language development depends heavily on sustained participation, interaction, and willingness to engage with linguistic input and classroom tasks (Lin and Wang, 2025; Wang X. et al., 2025; Wang and Xue, 2024). In second language research, engagement has also been discussed as learners' engagement with language itself, highlighting how learners attend to and work with language during learning activities (Svalberg, 2009; Wang et al., 2025d). As a result, engagement is now widely regarded as a dynamic and meaningful indicator of learning quality rather than a simple measure of time spent on tasks (Zhang, 2021).

Relevant studies generally view classroom engagement as a construct with multiple dimensions (Sinatra et al., 2015). Most frameworks focus on three main dimensions: behavioral, emotional, and cognitive engagement. Behavioral engagement refers to learners' observable participation, effort, and persistence in classroom activities; emotional engagement describes learners' feelings toward learning such as interest, enjoyment, or anxiety; cognitive engagement reflects learners' mental investment in learning, including the use of learning strategies and self-regulation (Fredricks et al., 2004, 2016).

Although classroom engagement reflects learners' active involvement in learning, it is not only a personal trait but is also shaped by contextual and motivational factors. From an SDT perspective, classroom engagement is more likely to develop when learners' BPNs for autonomy, competence, and relatedness are satisfied (Deci and Ryan, 2000). When these needs are supported, learners tend to show greater effort, more positive learning-related emotions, and deeper cognitive involvement (Ryan and Deci, 2017). In AI-assisted EFL learning contexts, classroom engagement is closely related to how learners perceive and use AI tools during learning (Guo and Wang, 2025; Wang et al., 2025b; Wu et al., 2025). Emerging empirical studies suggest that AI-related beliefs, such as AI trust and AI self-efficacy, are associated with learners' willingness to persist, invest effort, and engage more actively in AI-supported language learning tasks (Tram et al., 2024; Wang and Wang, 2025; Yu et al., 2026). However, despite the growing use of AI in EFL classrooms, research that explicitly links AI-related beliefs to classroom engagement remains limited, indicating the need for further investigation.

### 2.3 Basic psychological needs satisfaction

SDT argues that learners are more likely to show high-quality motivation when three BPNs are satisfied: autonomy, competence, and relatedness (Deci and Ryan, 2000). Autonomy is the sense of having choice and control over one's learning actions, competence is the sense of being capable and making progress, and relatedness is the sense of being connected and respected by others (Ryan and Deci, 2017, 2020). When these needs are met, learners tend to learn with more interest and persistence, which provides a clear motivational basis for classroom engagement.

In AI-assisted EFL learning, BPNs satisfaction reflects whether learners feel that AI-supported learning offers them meaningful choice, helps them make progress, and provides a sense of encouragement or connection (Ryan and Deci, 2017). Research on AI-supported EFL learning suggests that flexible pacing and targeted feedback are often associated with autonomy and competence satisfaction, while relatedness is more likely when AI interaction is perceived as responsive and when teachers guide AI use in supportive ways (Annamalai et al., 2023; Chiu et al., 2023; Wu and Wang, 2025). At the same time, these benefits depend on instructional design: learners may feel less autonomous or competent if AI use is experienced as controlling or poorly integrated into classroom activities (Li et al., 2025; Wang et al., 2025b; Wang and Guo, 2026).

Overall, empirical studies in AI-mediated language learning show a consistent pattern: when learners experience higher satisfaction of autonomy, competence, and relatedness, they also report higher engagement and stronger motivation to continue AI-supported learning (Annamalai et al., 2025; Chiu et al., 2024; Li and Chiu, 2025). From an SDT perspective, this pattern suggests that BPN satisfaction functions as a key motivational process through which learners' perceptions and experiences in AI-supported learning are translated into active engagement (Wang and Wang, 2024). In AI-assisted EFL contexts, learners' confidence in using AI and their willingness to rely on AI support can be understood as important conditions that shape whether competence and autonomy needs are satisfied, which in turn supports sustained classroom engagement (Li et al., 2025).

### 2.4 AI trust

In AI-assisted EFL learning, AI trust refers to learners' willingness to rely on AI systems for language-related support, such as feedback, explanations, or suggestions (Wang F. et al., 2025). Rather than being limited to technical reliability, recent research conceptualizes AI trust as a multifaceted belief that includes perceived competence and accuracy, perceived transparency, and perceived appropriateness of AI use in educational contexts (Jiang et al., 2025; Mehrotra et al., 2024; Pitts and Motamedi, 2025). This broader view is particularly relevant in EFL learning, where students often need to decide whether to accept, adapt, or question AI-generated language output during complex tasks such as writing or revision (Wang, 2026). From a learning perspective, AI trust is closely related to sustained engagement with AI-supported activities, as learners are more likely to invest effort and attention when they perceive AI support as reliable and appropriate (Hou et al., 2025; Wang F. et al., 2025; Wang et al., 2025c). At the same time, AI trust is shaped by both system features and classroom task design and overly low or overly high levels of trust may hinder effective engagement in AI-assisted EFL learning (Khoso et al., 2026; Wang et al., 2026; Zhang et al., 2025).

### 2.5 AI self-efficacy

Self-efficacy refers to individuals' beliefs about their capability to perform actions required to achieve desired outcomes in specific situations (Bandura, 1977). In AI-supported learning, this idea has been extended to AI self-efficacy, which reflects learners' confidence in their ability to use and interact with AI systems effectively for learning purposes. Recent work defines AI self-efficacy as learners' perceived capability to understand, operate, and benefit from AI tools during task completion, rather than merely possessing technical skills (Wang and Chuang, 2024). Empirical evidence across educational domains suggests that AI self-efficacy plays a meaningful role in shaping learners' attitudes, persistence, and engagement in AI-supported learning (Chen et al., 2024; Shao et al., 2025; Wang and Gao, 2025). In EFL contexts, AI self-efficacy is particularly relevant because learners must actively interpret, evaluate, and integrate AI-generated feedback into their own language production. From a motivational perspective, learners who feel capable of using AI are more likely to invest effort and remain engaged when AI becomes part of classroom learning, making AI self-efficacy a plausible psychological antecedent of sustained engagement in AI-assisted EFL settings (Huang et al., 2024; Wang and Chuang, 2024; Wang et al., 2025b).

### 2.6 The hypothesized model

Building on the conceptual and empirical insights discussed above, classroom engagement in AI-assisted EFL learning is conceptualized as a motivational outcome shaped by learners' AI-related beliefs and their BPNs satisfaction. Previous studies have shown that classroom engagement reflects learners' sustained behavioral, emotional, and cognitive involvement and is strongly influenced by motivational and contextual factors (Fredricks et al., 2016; Reeve, 2013; Skinner and Pitzer, 2012). From an SDT perspective, engagement is more likely to emerge when learners' needs for autonomy, competence, and relatedness are satisfied (Deci and Ryan, 2000; Ryan and Deci, 2017). In AI-assisted EFL contexts, learners' AI trust and AI self-efficacy have been shown to shape how AI-supported learning is perceived and experienced, particularly in terms of competence and autonomy support (Annamalai et al., 2023; Chiu et al., 2023; Li et al., 2025; Wang and Chuang, 2024). At the same time, accumulating evidence suggests that BPNs satisfaction functions as a more proximal motivational mechanism underlying classroom engagement than AI-related beliefs alone (Ryan and Deci, 2017; Wang and Wang, 2024).

**Figure 1:**
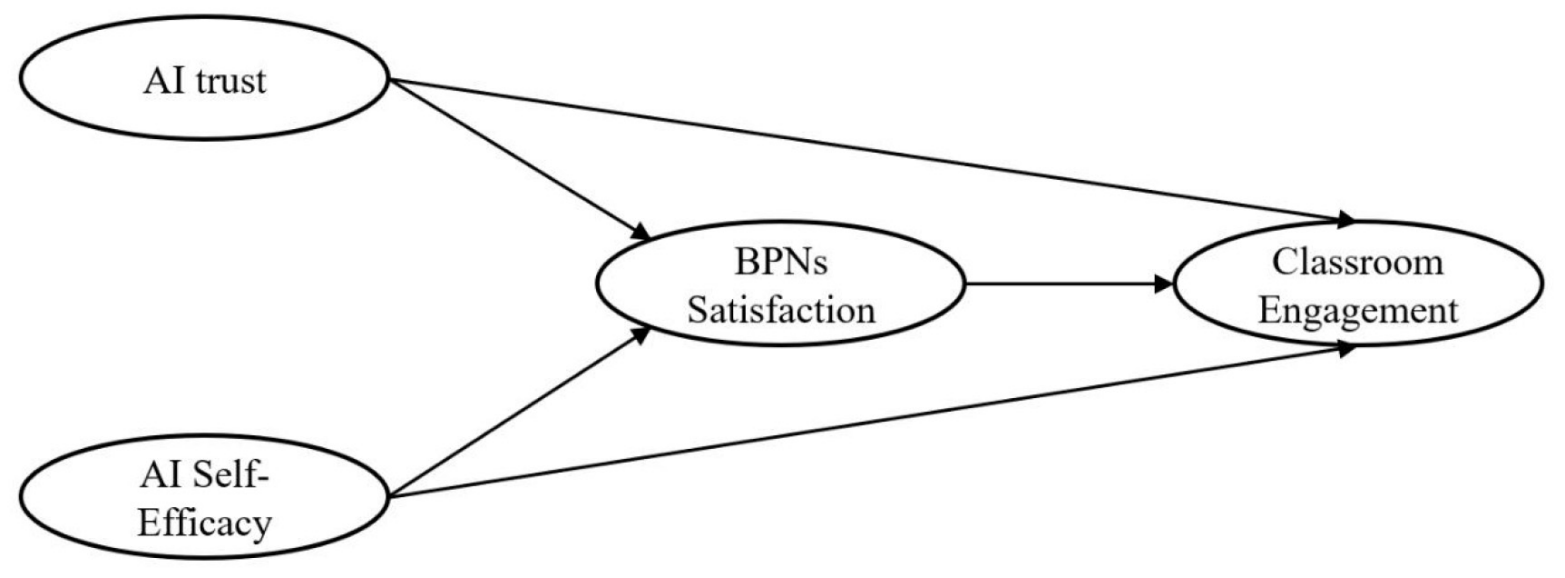
The hypothesis model.

Accordingly, the following hypotheses are proposed (see Figure 1):

H1: AI trust is positively associated with learners' BPNs satisfaction

H2: AI self-efficacy is positively associated with learners' BPNs satisfaction

H3: BPNs satisfaction is positively associated with classroom engagement.

H4: AI trust is positively associated with classroom engagement.

H5: AI self-efficacy is positively associated with classroom engagement.

H6: BPNs satisfaction mediates the relationship between AI trust and classroom engagement.

H7: BPNs satisfaction mediates the relationship between AI self-efficacy and classroom engagement.

## 3 Methodology

### 3.1 Participants

The participants were 1,002 undergraduate students from a comprehensive university in China (see Table 1). Among them, 457 were male (45.61%) and 545 were female (54.39%). In terms of age, 206 students (20.56%) were 18 years old or younger, 682 students (68.06%) were between 19 and 20 years old, 71 students (7.09%) were aged 21–22, and 43 students (4.29%) were 23 years old or above. With regard to year of study, the sample included 440 first-year students, 450 second-year students, 46 third-year students, and 66 fourth-year students. All participants were enrolled in required EFL courses at the time of data collection and had prior experience using AI-based tools as part of their classroom.

**Table 1:** Demographic information.

Variable	Category	*n*	%
Gender	Male	457	45.61
	Female	545	54.39
Age	18 or below	206	20.56
	19–20	682	68.06
	21–22	71	7.09
	23 or above	43	4.29
Year of study	First year	440	43.91
	Second year	450	44.91
	Third year	46	4.59
	Fourth year	66	6.59

### 3.2 Data collection

Data were collected through an online questionnaire administered via Wenjuanxing, a widely used survey platform in China. The questionnaire was distributed to students enrolled in the target EFL courses with the assistance of course instructors. Data collection took place over a 6-week period from December 2025 to January 2026.

At the beginning of the questionnaire, an informed consent statement was provided. The statement clearly explained the purpose of the study, the voluntary nature of participation, and the confidentiality of responses. Students were informed that their participation would not affect their course grades and that they could withdraw from the survey at any time without penalty. Only students who indicated their consent were allowed to proceed to the main questionnaire. All responses were collected anonymously, and no personally identifiable information was collected. The data were used solely for research purposes. The study was conducted in accordance with the relevant institutional and national ethical guidelines for research involving human participants.

To reduce the potential influence of common method bias, several procedural remedies were applied during data collection, including voluntary participation, anonymous responses, and clear instructions stating that there were no right or wrong answers (Podsakoff et al., 2003). In addition, a post hoc statistical assessment of common method variance was conducted and is reported in the Results section.

### 3.3 Instruments

All instruments used in this study were adapted from previously validated English-language scales. Following standard cross-cultural research procedures, the original English questionnaires were translated into Chinese. The translated versions were then reviewed by two experts with experience in EFL research and educational measurement to ensure linguistic accuracy, conceptual equivalence, and appropriateness for the AI-assisted EFL classroom context. Any discrepancies were discussed and resolved through consensus before finalizing the questionnaire. All items across the four scales were measured using a five-point Likert scale, ranging from 1 (strongly disagree) to 5 (strongly agree).

AI trust was measured using a three-item scale developed by McGrath et al. (2025), which assesses learners' willingness to rely on AI systems and their perceptions of AI reliability in task support. In the present study, the scale demonstrated good internal consistency (Cronbach's α = 0.926). The present study used this validated short scale to capture learners' overall trust in AI for classroom task support, rather than to differentiate among specific subdimensions such as transparency, appropriateness, or perceived competence.

AI self-efficacy was assessed using the six-item GSE-6AI scale adapted by Morales-García et al. (2024). This scale focuses on learners' perceived capability to use AI tools effectively for learning purposes. The internal consistency of the scale in this study was high (Cronbach's α = 0.960).

Learners' BPNs satisfaction was measured using a nine-item scale developed by Hew and Kadir (2016) grounded in self-determination theory. The scale captures learners' perceived satisfaction of autonomy, competence, and relatedness in technology-supported learning environments. The scale showed good internal consistency in the current sample (Cronbach's α = 0.981).

Classroom engagement was measured using the nine-item Language Classroom Engagement Scale (LCES) developed by Eerdemutu et al. (2024). The scale assesses learners' behavioral, emotional, and cognitive engagement in language classroom activities. In this study, the scale demonstrated good internal consistency (Cronbach's α = 0.976).

### 3.4 Data analysis

All statistical analyses were conducted using IBM SPSS AMOS 24.0. Data screening was performed prior to model testing to ensure that the dataset was suitable for structural equation modeling. The analysis followed a two-step procedure. First, a confirmatory factor analysis (CFA) was conducted to evaluate the measurement model, including the factor structure of the four latent constructs (AI trust, AI self-efficacy, BPNs satisfaction, and classroom engagement). Model fit was evaluated using commonly reported fit indices in SEM research. Standardized factor loadings were also examined to assess the strength of the relationships between items and their respective latent constructs.

Reliability and convergent/discriminant validity were examined based on the CFA results. Internal consistency was assessed using Cronbach's alpha. Composite reliability (CR) and average variance extracted (AVE) were calculated to evaluate construct reliability and convergent validity. Discriminant validity was first assessed using the Fornell-Larcker criterion by comparing the square root of AVE for each construct with its correlations with other constructs (Fornell and Larcker, 1981). In addition, the heterotrait–monotrait ratio (HTMT) was calculated as a more stringent test of discriminant validity (Henseler et al., 2015).

To assess the potential influence of common method variance, a CFA-based single-factor model was also tested by loading all measurement items onto one latent factor and comparing its fit with that of the hypothesized four-factor measurement model (Podsakoff et al., 2003).

After establishing an acceptable measurement model, structural equation modeling (SEM) was conducted to examine the hypothesized relationships among AI trust, AI self-efficacy, BPNs satisfaction, and classroom engagement. Direct relationships were estimated according to the proposed model, and the mediating role of BPNs satisfaction was examined within the SEM framework.

## 4 Results

### 4.1 Preliminary analyses

Table 2 presents the descriptive statistics of the four constructs. The mean values ranged from 3.923 to 4.102, indicating that participants generally reported moderately high levels of AI trust, AI self-efficacy, BPNs satisfaction, and classroom engagement. The skewness and kurtosis values were small and within acceptable ranges, suggesting that no severe departures from normality were observed at the construct level.

**Table 2:** Descriptive statistics.

Construct	Mean	SD	Kurtosis	Skewness
AI trust	3.923	0.716	−0.013	−0.137
AI self-efficacy	3.976	0.718	0.115	−0.233
BPNs satisfaction	4.102	0.665	0.012	−0.267
Classroom engagement	4.086	0.696	−0.291	−0.235

Table 3 summarizes the fit indices of the hypothesized four-factor measurement model and the single-factor model used for the assessment of common method variance. The results indicated an acceptable fit to the data (RMSEA = 0.079, CFI = 0.949, NFI = 0.942, IFI = 0.949, TLI = 0.944), meeting commonly recommended benchmark values (Kline, 2016). On this basis, the measurement model was considered adequate, and the structural model was subsequently tested to examine the hypothesized relationships.

**Table 3:** Fit indices for the four-factor and single-factor models.

Model	RMSEA	CFI	NFI	IFI	TLI
Four-factor	0.079	0.949	0.942	0.949	0.944
Single-factor	0.182	0.727	0.721	0.727	0.704
Benchmark value	< 0.08	> 0.90	> 0.90	> 0.90	> 0.90

By contrast, the single-factor model showed a substantially poorer fit to the data (RMSEA = 0.182, CFI = 0.727, NFI = 0.721, IFI = 0.727, TLI = 0.704). Compared with the hypothesized four-factor measurement model, this markedly poorer fit suggests that a single common factor could not adequately account for the covariance among the study variables. Therefore, common method variance was unlikely to fully account for the observed relationships in the present study (Podsakoff et al., 2003).

Next, as reported in Table 4, standardized factor loadings ranged from 0.863 to 0.944, indicating that all items loaded strongly on their intended constructs. CR and AVE were also calculated, and all values met commonly accepted criteria for construct reliability and convergent validity. Discriminant validity was evaluated using the Fornell-Larcker criterion. The square roots of AVE (reported on the diagonal) were greater than the correlations between constructs, indicating adequate discriminant validity (Fornell and Larcker, 1981).

**Table 4:** Reliability and discriminant validity (Fornell-Larcker criterion).

Construct	Loading range	AVE	CR	1	2	3	4
AI trust	0.881–0.923	0.806	0.926	**0.898**			
AI self-efficacy	0.863–0.934	0.802	0.960	0.856	**0.896**		
BPNs satisfaction	0.905–0.944	0.852	0.972	0.770	0.770	**0.923**	
Classroom engagement	0.872–0.925	0.816	0.976	0.646	0.669	0.849	**0.903**

Bold diagonal elements represent the square roots of AVE.

In addition, discriminant validity was further examined using the HTMT, which provides a more stringent assessment. As shown in Table 5, all HTMT values ranged from 0.644 to 0.856, which are below the recommended threshold of 0.90, further supporting the discriminant validity of the measurement model (Henseler et al., 2015).

**Table 5:** HTMT of construct correlations.

Construct	1	2	3	4
AI trust	–			
AI self-efficacy	0.856	–		
BPNs satisfaction	0.663	0.644	–	
Classroom engagement	0.772	0.770	0.849	–

### 4.2 Structural model results

**Figure 2:**
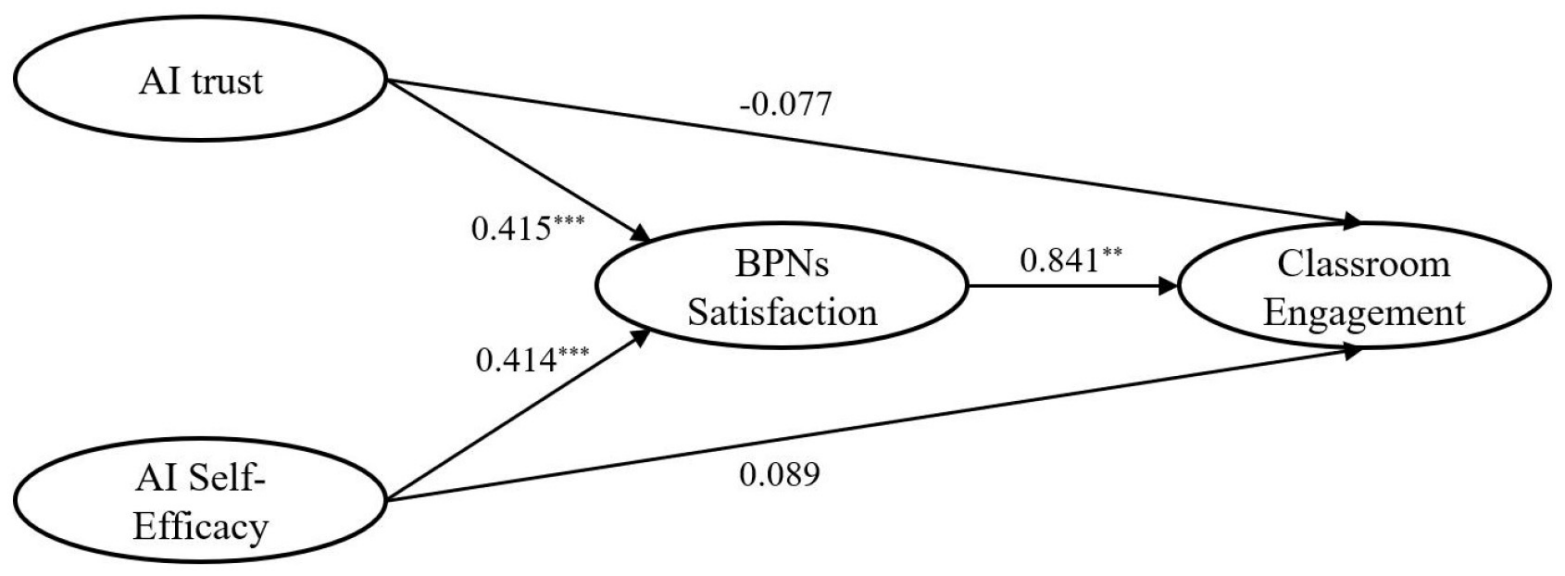
Structural equation model. Model fit: χ^2^_(317)_ = 2320.887, *p* < 0.001, χ^2^/df = 7.321, SRMR =0.026, RMSEA = 0.079, CFI = 0.949, NFI = 0.942, IFI = 0.949, TLI = 0.944.

The structural equation model was tested to examine the hypothesized relationships among AI trust, AI self-efficacy, BPNs satisfaction, and classroom engagement. Figure 2 presents the standardized path coefficients of the structural model, and Table 6 summarizes the direct and indirect effects with corresponding confidence intervals.

**Table 6:** Structural model results for direct and indirect effects.

Hypothesis	Path	β	SE	95% CI	*p*	Results
Direct effect						
H1	AI trust → BPNs satisfaction	0.415	0.066	(0.291, 0.549)	< 0.001	Supported
H2	AISE → BPNs satisfaction	0.414	0.066	(0.282, 0.541)	< 0.001	Supported
H3	BPNs satisfaction → CE	0.841	0.043	(0.739, 0.911)	0.001	Supported
H4	AI Trust → CE	−0.077	0.054	(−0.187, 0.025)	0.138	Not supported
H5	AISE → CE	0.089	0.050	(−0.005, 0.194)	0.065	Not supported
Indirect effect						
H6	AI trust → BPNs satisfaction → CE	0.349	—	(0.239, 0.474)	< 0.001	Supported
H7	AISE → BPNs satisfaction → CE	0.348	—	(0.235, 0.460)	< 0.001	Supported

AISE, AI self-efficacy; CE, classroom engagement.

Regarding direct effects, AI trust was positively associated with BPNs satisfaction [β = 0.415, SE = 0.066, 95% CI (0.291, 0.549), *p* < 0.001], supporting H1. Similarly, AI self-efficacy showed a significant positive relationship with BPNs satisfaction [β = 0.414, SE = 0.066, 95% CI (0.282, 0.541), *p* < 0.001], supporting H2. BPNs satisfaction was strongly and positively related to classroom engagement [β = 0.841, SE = 0.043, 95% CI (0.739, 0.911), *p* = 0.001], providing support for H3.

In contrast, the direct effects of AI trust and AI self-efficacy on classroom engagement were not statistically significant. The path from AI trust to classroom engagement was negative and non-significant [β = −0.077, SE = 0.054, 95% CI (−0.187, 0.025), *p* = 0.138], and the path from AI self-efficacy to classroom engagement was also non-significant [β = 0.089, SE = 0.050, 95% CI (−0.005, 0.194), *p* = 0.065]. Therefore, H4 and H5 were not supported.

With respect to indirect effects, both AI self-efficacy and AI trust showed significant indirect relationships with classroom engagement through BPNs satisfaction. The indirect effect of AI self-efficacy on classroom engagement via BPNs satisfaction was significant [β = 0.348, 95% CI (0.235, 0.460), *p* < 0.001], supporting H6. Similarly, the indirect effect of AI trust on classroom engagement through BPNs satisfaction was significant [β = 0.349, 95% CI (0.239, 0.474), *p* < 0.001], supporting H7. These results indicate that BPNs satisfaction statistically mediated the relationships between AI-related beliefs and classroom engagement in the proposed model.

## 5 Discussion

The present study examined the relationships among AI trust, AI self-efficacy, BPNs satisfaction, and classroom engagement in an AI-assisted EFL context. The results reveal a clear and theoretically coherent pattern. Both AI trust and AI self-efficacy were positively associated with BPNs satisfaction, and BPNs satisfaction, in turn, showed a strong positive association with classroom engagement. However, the direct effects of AI trust and AI self-efficacy on classroom engagement were not significant once BPNs satisfaction was included in the model. Instead, both variables showed significant indirect associations with engagement through BPNs satisfaction. These findings suggest that learners' AI-related beliefs are related to classroom engagement mainly through their associations with motivational experiences, rather than through direct relationships with engagement behaviors. This pattern is also consistent with the central propositions of SDT (Deci and Ryan, 2000; Ryan and Deci, 2017), which posit that contextual and belief-related factors influence engagement primarily through their effects on the satisfaction of learners' basic psychological needs for autonomy, competence, and relatedness. In this sense, AI trust and AI self-efficacy may function as antecedent perceptions that shape learners' motivational experiences, while BPNs satisfaction represents the more proximal mechanism that energizes classroom engagement.

The positive associations between AI trust, AI self-efficacy, and BPNs satisfaction are consistent with previous studies. In AI-assisted EFL contexts, learners who perceive AI tools as reliable and useful tend to experience learning tasks as more manageable and supportive, particularly with regard to competence and autonomy (Annamalai et al., 2023; Chiu et al., 2023; Li et al., 2025). Similarly, learners with higher AI self-efficacy are more likely to feel capable of interacting with AI-generated feedback and suggestions, which can reduce uncertainty and support a sense of progress during task completion (Wang and Chuang, 2024; Wang and Wang, 2024; Wang et al., 2025b). Together, these findings suggest that positive beliefs about AI are associated with more need-supportive learning experiences in AI-assisted EFL classrooms. However, treating BPNs satisfaction as a single latent construct reduces the theoretical granularity of the findings. From an SDT perspective, autonomy, competence, and relatedness are distinguishable needs (Deci and Ryan, 2000; Ryan and Deci, 2017). Thus, although the present model captures the overall motivational role of BPNs satisfaction, it does not reveal whether AI trust and AI self-efficacy are linked to classroom engagement through different need-based pathways (Annamalai et al., 2023; Chiu et al., 2023).

The strong association between BPNs satisfaction and classroom engagement further supports self-determination theory and prior engagement research. Engagement has been widely conceptualized as a motivationally driven and context-sensitive process, rather than a stable learner characteristic (Reeve, 2013; Skinner and Pitzer, 2012; Zhang, 2021). In EFL classrooms, engagement reflects learners' sustained behavioral participation, emotional involvement, and cognitive investment in language-related activities (Fredricks et al., 2016; Svalberg, 2009). Previous studies have shown that when learners feel autonomous, competent, and socially supported, they are more likely to remain engaged and persist in classroom learning (Li et al., 2025; Lin and Wang, 2025; Wu and Wang, 2025). The present findings are consistent with this line of research in AI-assisted settings and highlight the potential importance of motivational experiences in relation to classroom engagement.

The negative but non-significant direct path from AI trust to classroom engagement warrants a more cautious interpretation. Importantly, AI trust was positively correlated with classroom engagement at the bivariate level, but its direct coefficient became slightly negative after BPNs satisfaction and AI self-efficacy were included in the structural model. This suggests that the coefficient should be interpreted as a residual direct effect rather than as evidence that AI trust is inherently detrimental to classroom engagement. From an SDT perspective, the motivationally beneficial component of AI trust may be largely transmitted through learners' satisfaction of autonomy, competence, and relatedness, which are more proximal antecedents of engagement (Deci and Ryan, 2000; Ryan and Deci, 2017; Wang and Wang, 2024). Once this shared positive variance is taken into account, the remaining variance in AI trust may reflect a more passive reliance on AI, in which learners accept AI-generated suggestions without sufficient monitoring, evaluation, or reflection. In AI-assisted EFL classrooms, such reliance may weaken agentic and cognitive participation, especially when classroom tasks do not require learners to question, adapt, or meaningfully integrate AI output into their own learning processes (Reeve, 2013; Wang et al., 2025c; Yu et al., 2026; Zhang et al., 2025). At the same time, because this direct effect was not statistically significant, the negative direction should not be overinterpreted. A more appropriate conclusion is that AI trust appears to be associated with classroom engagement primarily through motivational experiences such as BPNs satisfaction, rather than through a stable direct pathway (Chiu et al., 2024; Li and Chiu, 2025; Wang and Wang, 2024).

The significant indirect effects observed in this study highlight the central role of BPNs satisfaction in the statistical relationships between AI-related beliefs and classroom engagement. The results suggest that AI trust and AI self-efficacy are associated with engagement primarily when they relate to learners' experiences of choice, competence, and connection within the classroom. This mediated pattern is consistent with prior findings in EFL and educational psychology research showing that motivational quality, rather than tool perception alone, underlies sustained engagement (Li et al., 2025; Ryan and Deci, 2017; Wang and Wang, 2024). In this sense, the present study complements earlier empirical work by clarifying how AI-related beliefs are situated within a broader motivational system, rather than treating them as direct predictors of engagement.

Overall, the findings suggest that AI trust and AI self-efficacy are variables associated with classroom engagement in AI-assisted EFL learning, with these relationships appearing to operate indirectly through learners' satisfaction of BPNs. By integrating AI-related beliefs into an SDT-based framework, this study contributes to understanding the relationships among learners' perceptions of AI, motivational experiences, and classroom engagement in formal classroom contexts. At the same time, these findings should be interpreted with caution, as the data were drawn from students in a single university context and were based on cross-sectional self-report measures. Therefore, the conclusions should not be generalized beyond similar educational settings without further empirical support.

## 6 Implications

The findings of this study suggest that promoting learners' trust in AI and their confidence in using AI is not sufficient by itself to ensure active classroom engagement. Instead, AI-related beliefs appear to influence engagement mainly through learners' satisfaction of basic psychological needs. This highlights the importance of focusing on the motivational quality of AI-supported instruction rather than treating AI adoption as a purely technical issue. In other words, the effectiveness of AI-assisted learning environments depends not only on the availability of AI tools but also on how these tools are integrated into learning activities in ways that support students' psychological needs.

At the classroom level, teachers should integrate AI tools in ways that support learners' autonomy, competence, and relatedness. For example, teachers can provide structured choices regarding how AI tools are used for language tasks, such as allowing students to select different AI-assisted strategies for drafting, revising, or practicing language skills. Such choices may enhance students' sense of autonomy and encourage them to take a more active role in their learning. In addition, competence support can be strengthened by making learning goals explicit and by providing guidance on how to interpret, evaluate, and apply AI-generated feedback. Rather than relying passively on AI outputs, students should be encouraged to critically engage with AI suggestions and integrate them into their own language production. Furthermore, designing AI-assisted activities that include peer interaction and teacher guidance may help maintain the social dimension of classroom learning and strengthen students' sense of relatedness within the learning environment.

At a broader level, the findings also suggest implications for teacher preparation and professional development related to AI integration in language education. Many current training initiatives focus primarily on the technical operation of AI tools. However, the results of this study indicate that teachers may also benefit from understanding how AI-supported instruction interacts with students' motivation and engagement. Professional development programs could therefore place greater emphasis on need-supportive instructional strategies, helping teachers design AI-assisted learning environments that foster autonomy, competence, and relatedness. By developing both technological and pedagogical awareness, educators may be better prepared to integrate AI in ways that support sustained engagement and meaningful learning in EFL classrooms.

## 7 Limitations and future directions

Several limitations of this study should be acknowledged. First, the data were collected using self-report questionnaires, which may be subject to common method bias and social desirability effects. Although validated instruments were used and the measurement model showed satisfactory psychometric properties, learners' reported engagement and AI-related beliefs may not fully capture their actual classroom behaviors. Future studies could incorporate multiple data sources, such as classroom observations, learning logs, or system-generated usage data, to provide a more comprehensive picture of engagement in AI-assisted EFL learning.

Second, the cross-sectional design limits causal interpretation of the relationships identified in this study. While the structural model was theoretically grounded, the directionality of the paths cannot be definitively established. Thus, the mediation model tested here should be interpreted as a theoretically informed statistical representation of the relationships among the study variables rather than as evidence of causal pathways. In addition, the present analysis focused on the hypothesized model derived from self-determination theory and did not test alternative competing models. Future research could therefore adopt longitudinal or intervention-based designs and compare alternative structural models to further examine the robustness of the proposed mediation structure.

Third, the sample was drawn from a single university context, which may limit the generalizability of the findings. Differences in institutional culture, instructional design, and AI integration practices may lead to different motivational dynamics. Future research could replicate the model across diverse educational contexts, including different universities, proficiency levels, or instructional formats. In addition, further studies may explore the distinct roles of autonomy, competence, and relatedness separately, as well as examine different dimensions of classroom engagement, to gain a more fine-grained understanding of motivational processes in AI-assisted EFL classrooms.

Finally, although basic psychological needs satisfaction was modeled as a single latent construct in the present study, SDT conceptualizes autonomy, competence, and relatedness as distinguishable dimensions. Future research may examine these three needs separately to provide a more fine-grained understanding of how each dimension is related to AI trust, AI self-efficacy, and classroom engagement in AI-assisted EFL contexts.

## Data Availability

The raw data supporting the conclusions of this article will be made available by the authors, without undue reservation.

## Appendices

**Appendix A**. Questionnaire items. The appendix presents the English-source/adapted item content for transparency; the questionnaire administered to participants was the Chinese translated version. All items were measured on a five-point Likert scale ranging from 1 (strongly disagree) to 5 (strongly agree). AI trust McGrath, M. J., Lack, O., Tisch, J., & Duenser, A. (2025). Measuring trust in artificial intelligence: validation of an established scale and its short form. *Frontiers in Artificial Intelligence, 8*, 1582880. https://doi.org/10.3389/frai.2025.1582880

1. I am confident in the AI assistant.
2. The AI assistant is reliable.
3. I can trust the AI assistant.

AI Self-efficacy Morales-García, W. C., Sairitupa-Sanchez, L. Z., Morales-García, S. B., & Morales-García, M. (2024). Adaptation and psychometric properties of a brief version of the general self-efficacy scale for use with artificial intelligence (GSE-6AI) among university students. *Frontiers in Education, 9*, 1293437. https://doi.org/10.3389/feduc.2024.1293437

1. If someone opposes me, I can find means and ways to get what I want by using artificial intelligence
2. It's easy for me to stay true to my goals and achieve my objectives with the help of artificial intelligence.
3. I am confident that I could efficiently face unexpected events by using artificial intelligence.
4. Thanks to my wit supported by artificial intelligence, I know how to handle unforeseen situations.
5. I can stay calm when facing difficulties because I trust in my coping skills backed by artificial intelligence.
6. No matter what comes up, I can usually handle it with the support of artificial intelligence.

Learners' BPNs satisfaction Hew, T. S., & Kadir, S. L. S. A. (2016). Understanding cloud-based VLE from the SDT and CET perspectives: Development and validation of a measurement instrument. *Computers & Education, 101*, 132-149. https://doi.org/10.1016/j.compedu.2016.06.004 Autonomy

1. I feel free to express my learning needs when communicating in English with the GenAI chatbot.
2. I feel relaxed and autonomous when engaging in English communication with the GenAI chatbot.
3. I have the opportunity to decide the conversation topics when interacting with the GenAI chatbot.

Competence

1. I experience a sense of achievement when communicating with the GenAI chatbot in English.
2. I feel capable when interacting with the GenAI chatbot in English.
3. I am satisfied with my English performance during conversations with the GenAI chatbot.

Relatedness

1. I feel supported during English conversations with the GenAI chatbot.
2. I feel understood when communicating in English with the GenAI chatbot.
3. I consider English interaction with the GenAI chatbot a meaningful experience.

Classroom engagement Eerdemutu, L., Dewaele, J. M., & Wang, J. (2024). Developing a short language classroom engagement scale (LCES) and linking it with needs satisfaction and achievement. *System, 120*, 103189. https://doi.org/10.1016/j.system.2023.103189

1. When I cannot understand in my language class, I stay focused until I do.
2. I put effort into learning in my language class.
3. I keep trying in my language class even if something is hard.
4. I look forward to my language class.
5. I enjoy learning new things about languages in class.
6. I feel good when I am in my language class.
7. In my language class, I think about different ways to solve a problem.
8. I try to connect what I am learning in the language classroom to what I have learned.
9. I try to understand my mistakes in the language classroom when I get something wrong.
